# Supplementary material for: The structural basis for 2′−5′/3′−5′-cGAMP synthesis by cGAS
Source: Nat Commun. 2024 May 13;15:4012. doi: 10.1038/s41467-024-48365-3 (PMC11091121; doi:10.1038/s41467-024-48365-3)
Supplement: Supplementary file 1 — Supplementary Information [file 41467_2024_48365_MOESM1_ESM.pdf]

Supporting information

Supplementary Figures 1-7

Supplementary Tables 1-3

Supplementary Figure 1

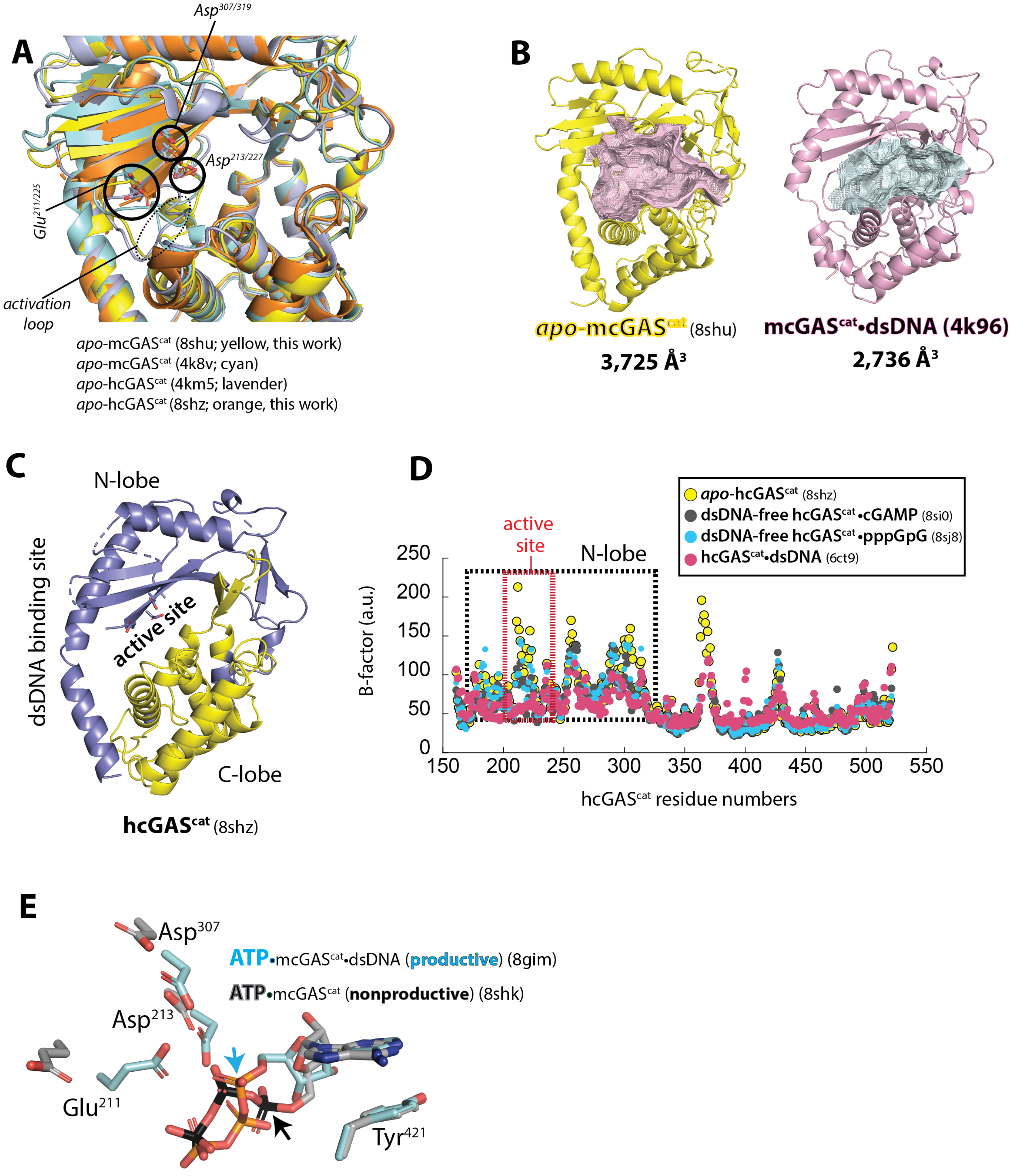

Supplementary Figure 1

(**A**) *apo*-cGAS^{cat} structures are aligned, which show multiple conformations at the active site.

(**B**) The active site cavity volume of dsDNA-free and dsDNA-bound mcGAS^{cat} (only the protein is shown for clarity); the volumes were calculated by CavityPlus [1].

(**C**) Two halves of hcGAS^{cat} surrounding the active site are colored differently.

(**D**) The B-factor of hcGAS^{cat} crystal structures at each position.

(**E**) An overlay of ATP-bound mcGAS^{cat} in the presence or absence of dsDNA. The positions of the target α-phosphate for the first linkage formation are indicated for both structures. The ribose of ATP bound to dsDNA-free mcGAS^{cat} was modeled without corresponding density for the presentation purpose only.

Supplementary Reference:

1. Xu, Y., Wang, S., Hu, Q., Gao, S., Ma, X., Zhang, W., Shen, Y., Chen, F., Lai, L., and Pei, J. (2018). CavityPlus: a web server for protein cavity detection with pharmacophore modelling, allosteric site identification and covalent ligand binding ability prediction. Nucleic Acids Res *46*, W374-W379. 10.1093/nar/gky380.

Supplementary Figure 2

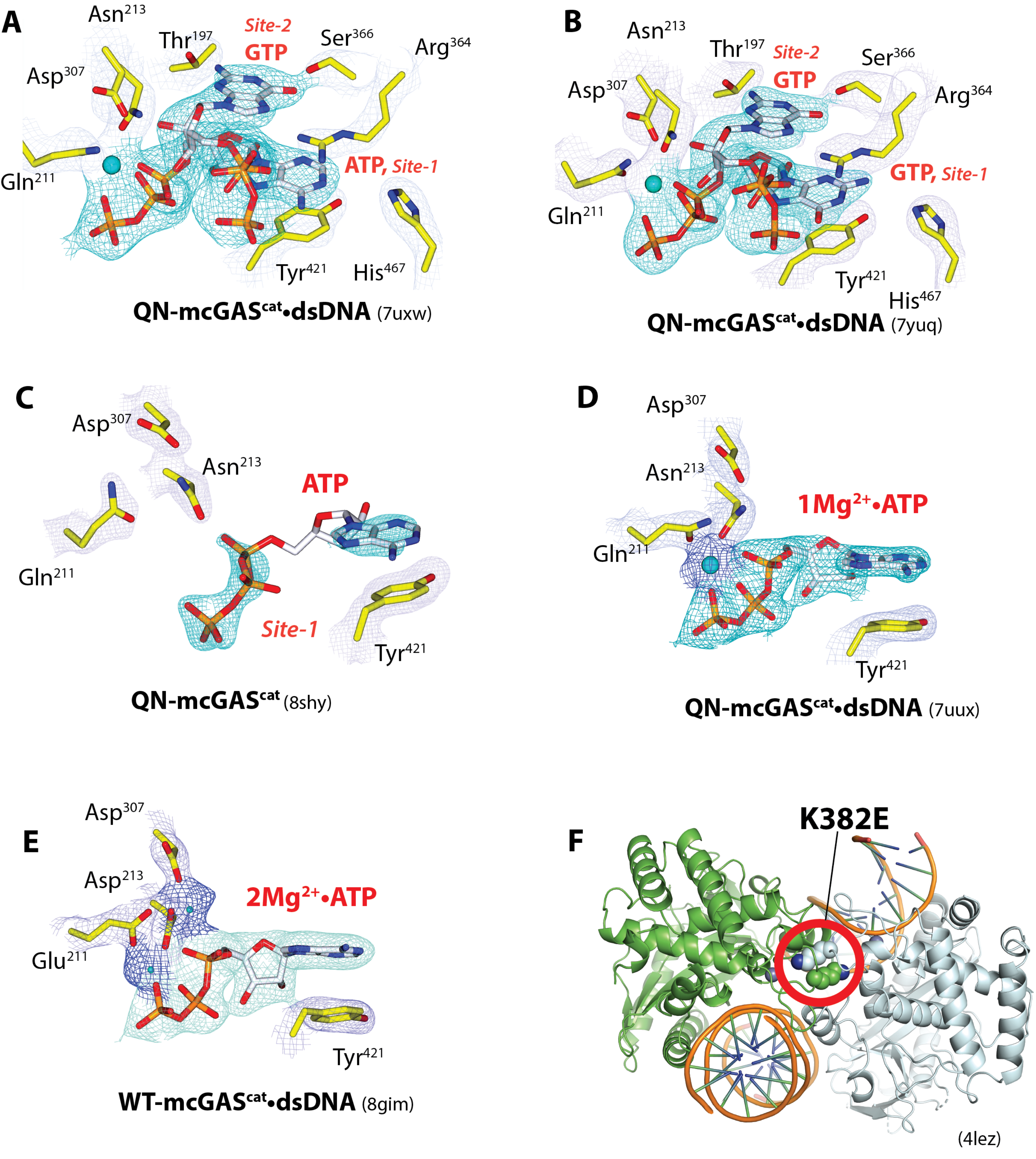

Supplementary Figure 2

(**A**) QN-mcGAS^{cat}•dsDNA bound to ATP and GTP showing corresponding electron densities (all 2Fo-Fc maps contoured at 1.5 σ hereafter).

(**B**) QN-mcGAS^{cat}•dsDNA bound to GTP and GTP showing corresponding electron densities.

(**C**) dsDNA-free QN-mcGAS^{cat} bound to ATP showing corresponding electron densities. Note the missing density for the ribose.

(**D**) QN-mcGAS^{cat}•dsDNA bound to ATP showing corresponding electron densities.

(**E**) WT-mcGAS^{cat}•dsDNA bound to ATP showing corresponding electron densities.

(**F**) The dimer interface (K382E) is indicated with the magenta circle.

Supplementary Figure 3

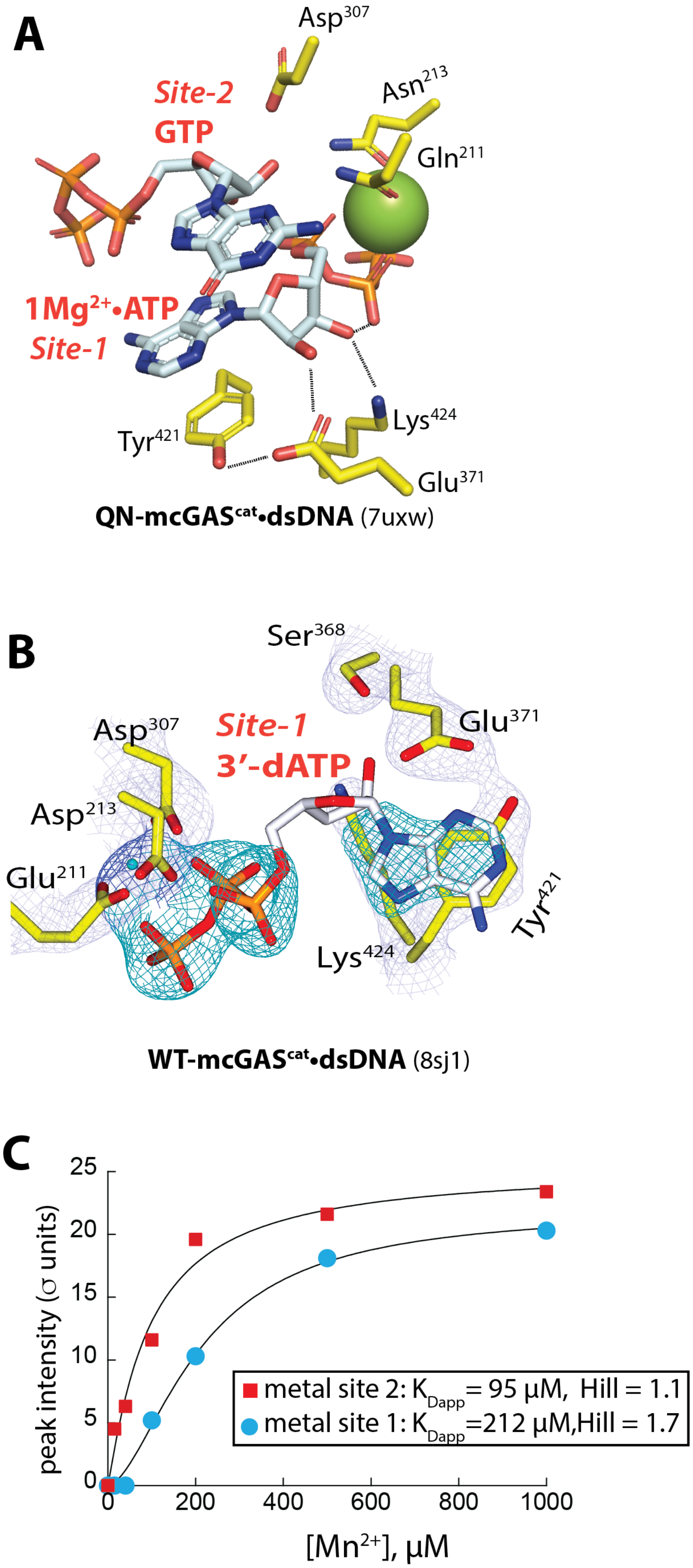

Supplementary Figure 3

(**A**) QN-mcGAS^{cat}•dsDNA bound to ATP and GTP. This "interior view" shows protein•NTP and intra-NTP interactions established by the 2'- and 3'-OH of ATP.

(**B**) WT-mcGAS^{cat}•dsDNA bound to 3'-dATP. The ribose was modeled without density.

(**C**) A pseudo-binding curve for Mn^{2+} affinity at each metal binding site based on the peak intensity (σ) in the anomalous map.

Supplementary Figure 4

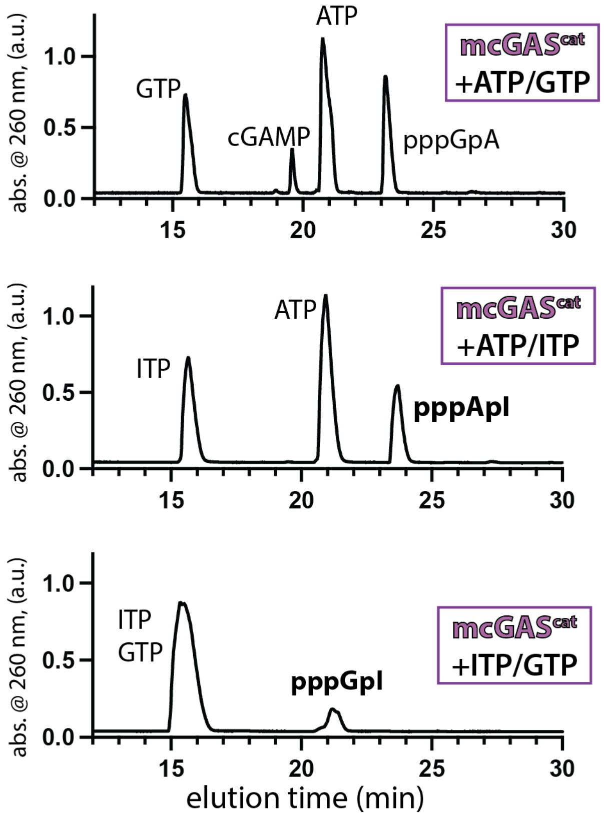

Supplementary Figure 4

Sample HPLC trace profiles of WT-mcGAS^{cat}•dsDNA reaction products resulting from ATP/GTP, ATP/ITP, and ITP/GTP (15 min reaction, 27 min gradient).

Supplementary Figure 5

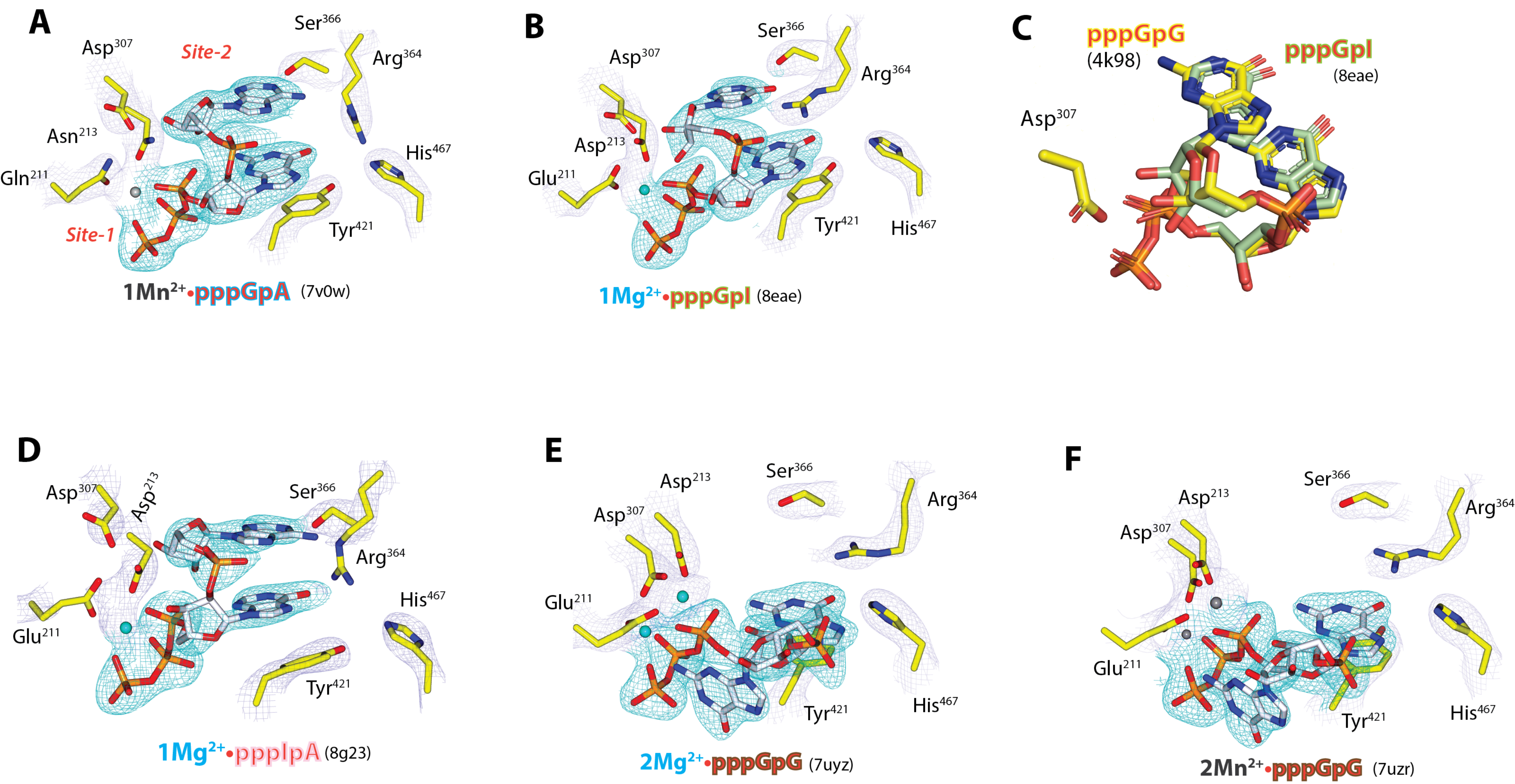

Supplementary Figure 5

(**A-B**) QN-mcGAS^{cat}•dsDNA bound to pppGpA showing corresponding electron densities. The presence of Mn^{2+} at Site-1 was tracked and confirmed via data collection at 1.8961Å

(**B**) WT-mcGAS^{cat}•dsDNA bound to pppGpI showing corresponding electron densities.

(**C**) An overlay of WT-mcGAS^{cat}•dsDNA bound to pppGpG (4K98 [https://doi.org/10.2210/pdb4K98/pdb]) and pppGpI

(**D-E**) WT-mcGAS^{cat}•dsDNA bound to pppIpA (**D**) and pppGpG (**E**) showing corresponding electron densities.

(**F**) WT-mcGAS^{cat}•dsDNA bound to 2Mn^{2+}•pppGpG showing corresponding electron densities.

Supplementary Figure 6

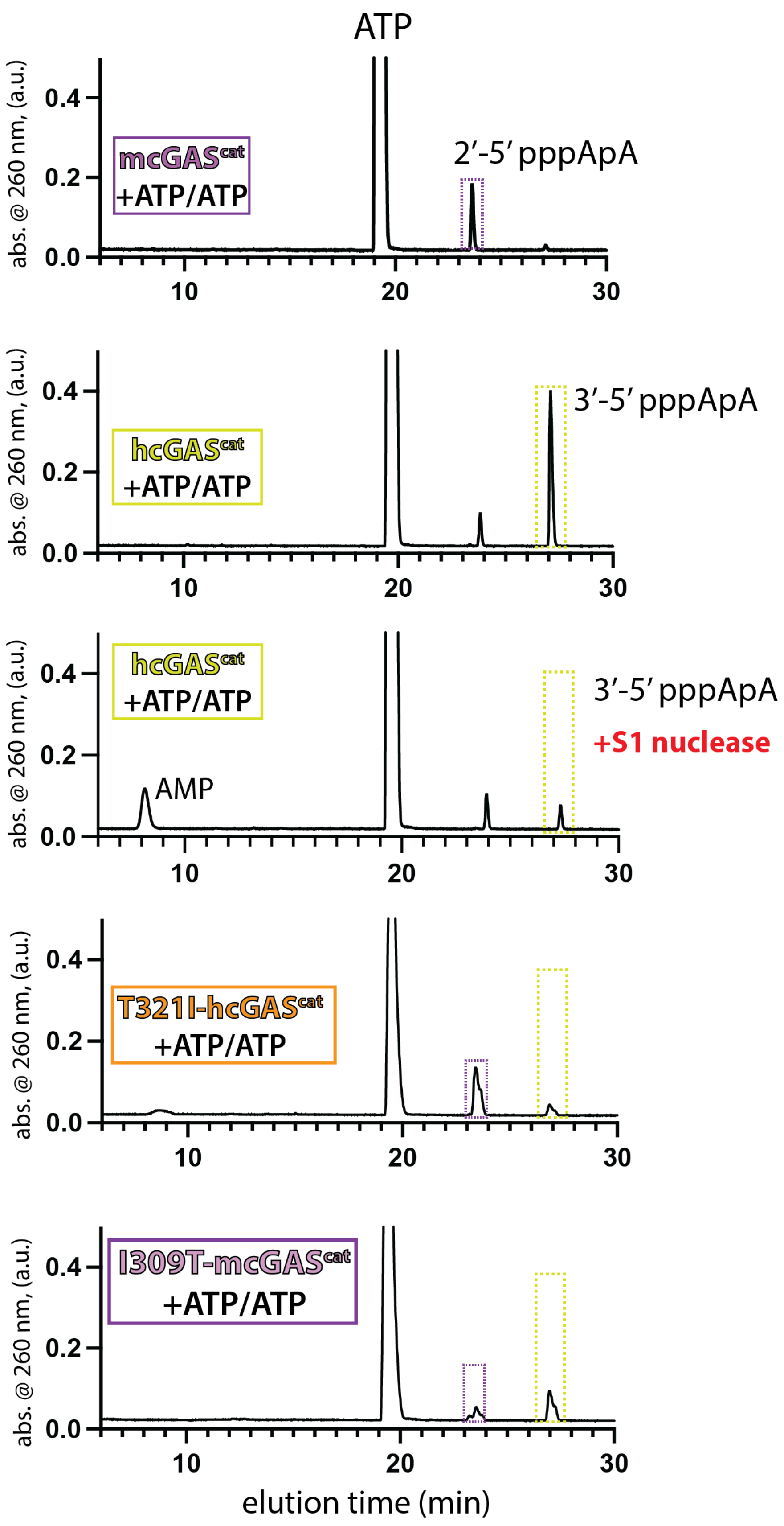

Supplementary Figure 6

Sample HPLC traces of reaction products resulting from WT and mutant h/mcGAS^{cat}•dsDNA against ATP/ATP (2 hr reaction, 27 min gradient). The S1 nuclease specifically degrades 3'-5'-linked oligonucleotides.

Supplementary Figure 7-1

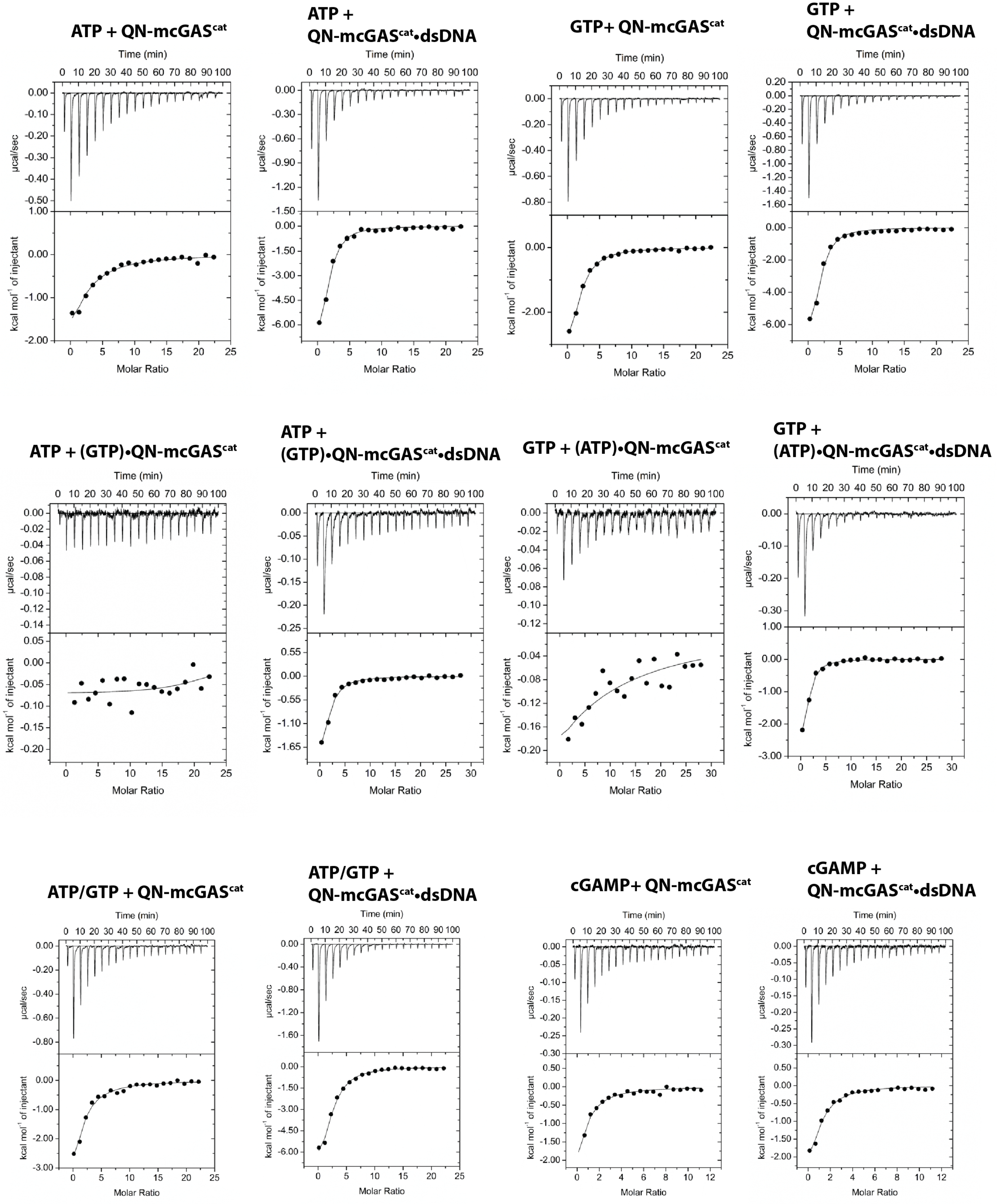

Supplementary Figure 7-2

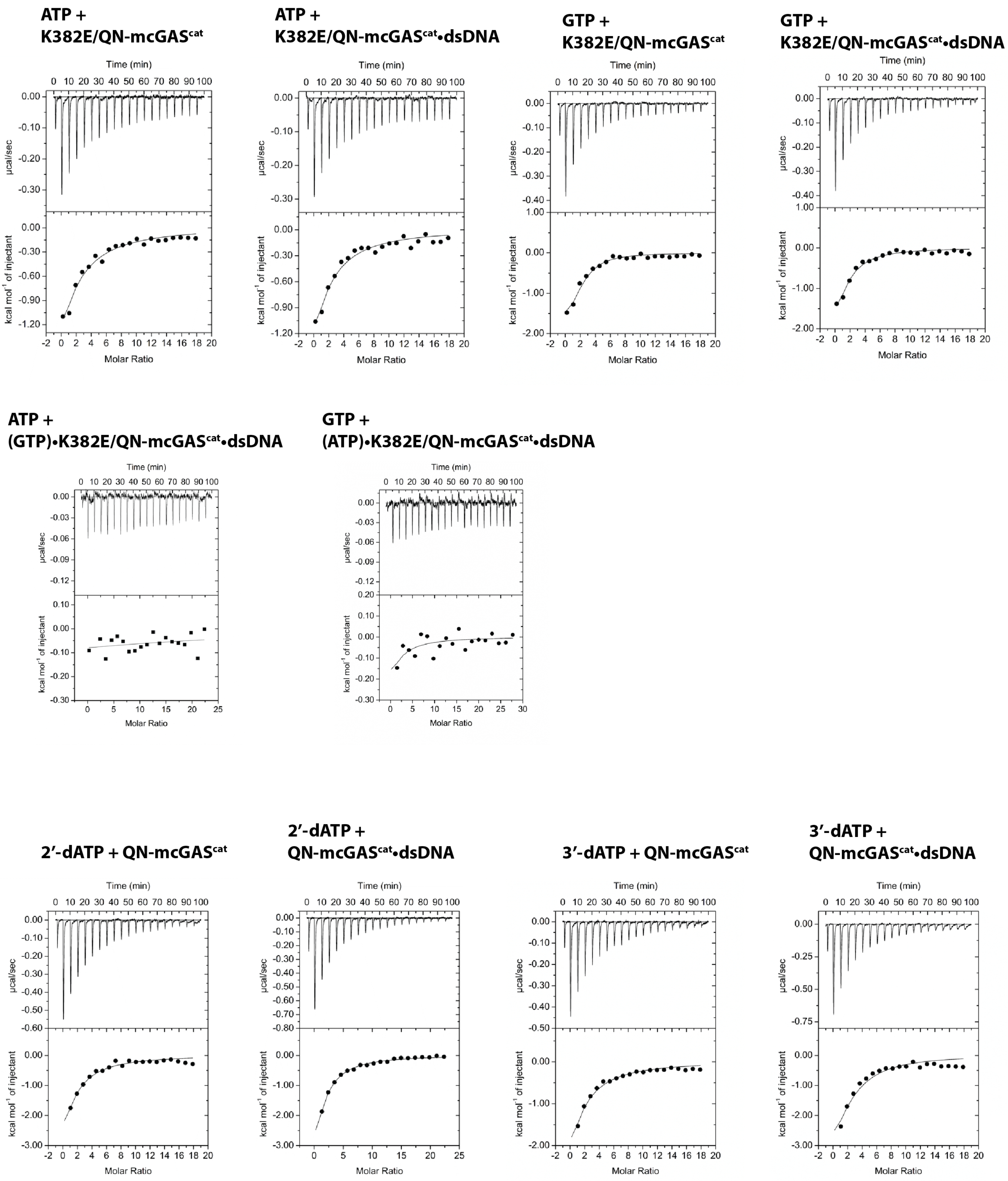

Supplementary Figure 7

Sample ITC profiles.

Supplementary Figure 8-1

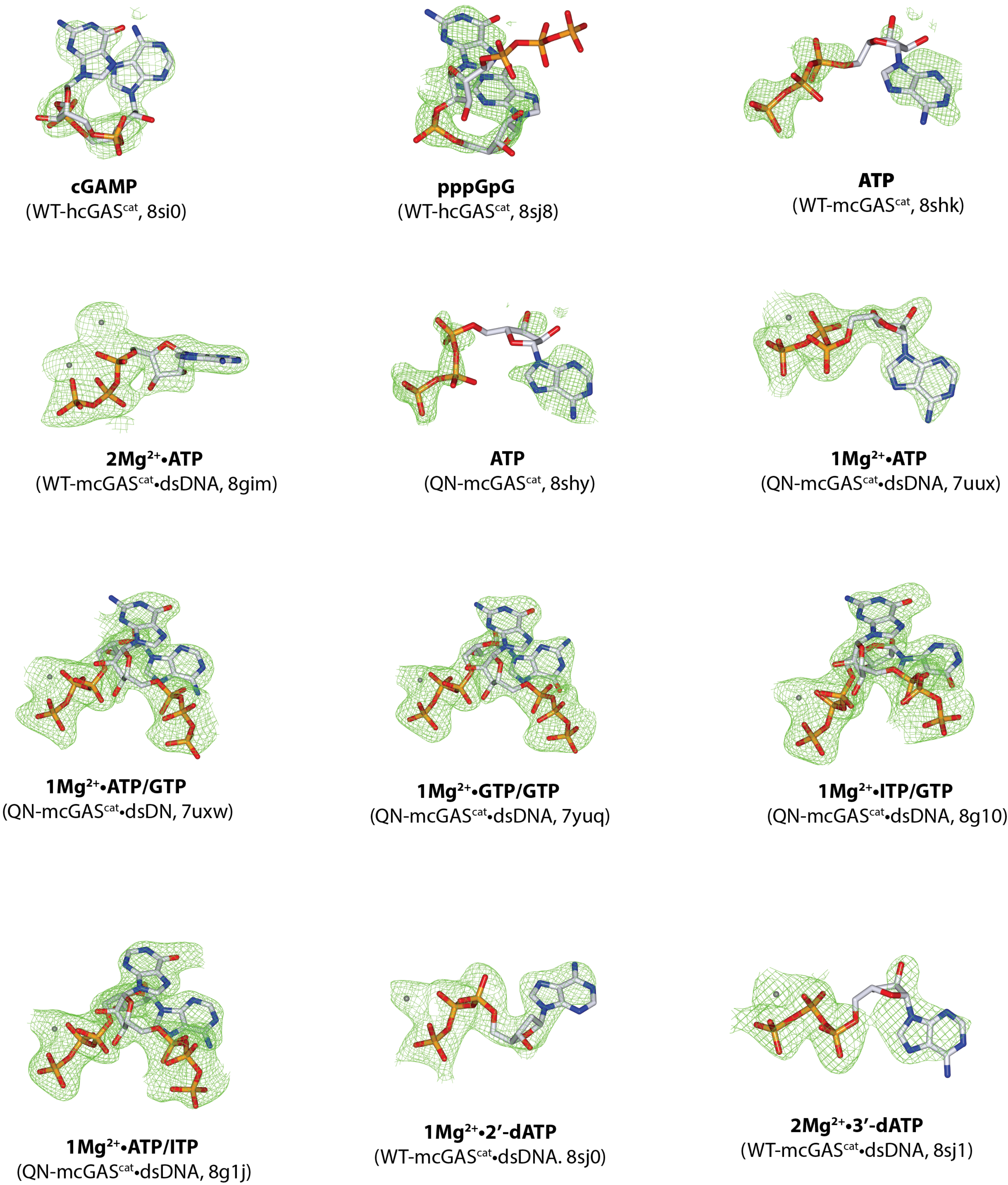

Supplementary Figure 8-2

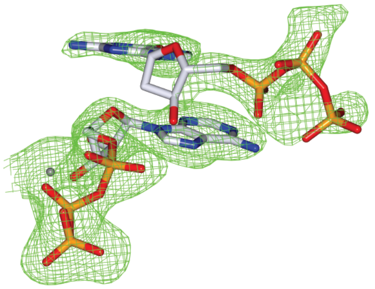

1Mg^{2+}•ATP/2′-dGTP
(WT-mcGAS^{cat}•dsDNA, 8sj2)

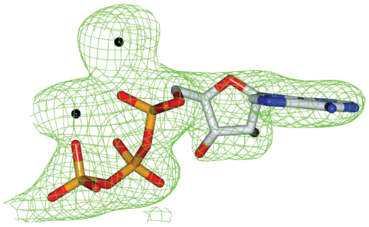

2Mn^{2+}•ATP
(WT-mcGAS^{cat}•dsDNA, 8skt)

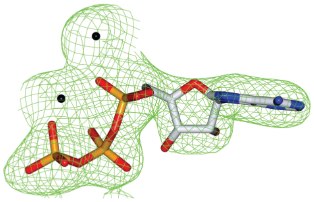

2Mn^{2+}•ATP
(WT-mcGAS^{cat}•dsDNA
1 mM Mn^{2+}/ 10 mM Mg^{2+}, 8git)

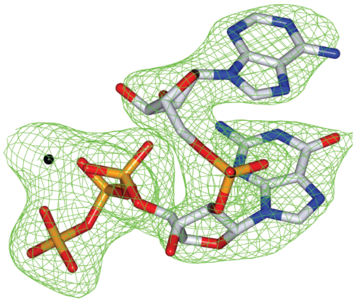

1Mn^{2+}•pppGpA
(QN-mcGAS^{cat}•dsDNA, 7v0w)

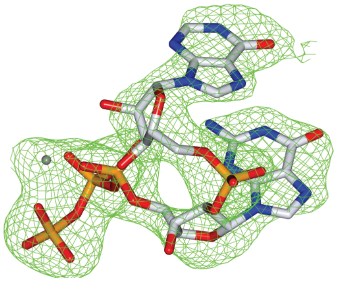

1Mg^{2+}•pppGpI
(WT-mcGAS^{cat}•dsDNA, 8eae)

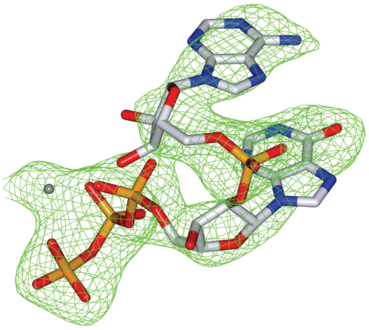

1Mg^{2+}•pppIpA
(WT-mcGAS^{cat}•dsDNA, 8g23)

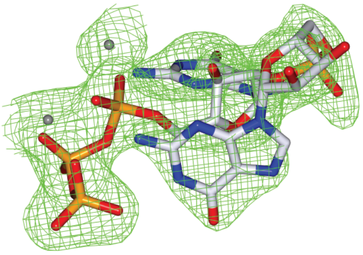

2Mg^{2+}•pppGpG
(WT-mcGAS^{cat}•dsDNA, 7uyz))

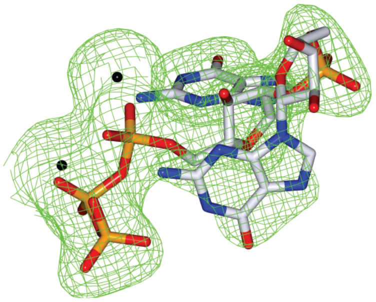

2Mn^{2+}•pppGpG
(WT-mcGAS^{cat}•dsDNA, 7uzr)

Supplementary Figure 8

Corresponding NTPs are modeled into the Fo-Fc (omit) map from each PDB entry (all contoured at 3σ).

Supplementary Table 1. X-ray Data Collection and Structure Refinement Statistics

**Crystal**	apo mcGAS WT	mcGAS WT+ATP	mcGAS QN+ATP
PDB code	8SHU	8SHK	8SHY
**Data collection**			
Beamline	17-ID-2 (FMX)	17-ID-1 (AMX)	17-ID-1 (AMX)
Wavelength (Å)	0.9793	0.9201	0.9201
Space group	I 1 2 1	I 1 2 1	I 1 2 1
Cell constants			
a, b, c (Å)	87.89, 61.05, 105.70	88.11, 61.23, 105.73	88.11, 60.97, 104.35
α, β, γ (°)	90.00, 94.57, 90.00	90.00, 94.36, 90.00	90.00, 94.59, 90.00
Resolution (Å)	28.74 -1.71 (1.77 -1.71) *	28.79 -1.71 (1.77 -1.71) *	27.94 -1.77 (1.83 -1.77) *
R_{merge}	0.036	0.079	0.098
I/σ (I)	10.64 (1.87)	10.82 (1.78)	8.19 (1.84)
Completeness (%)	97.75 (95.77)	99.03 (97.10)	99.30 (95.00)
Redundancy	3.3 (3.2)	3.5 (3.3)	3.5 (3.4)
**Refinement**			
No. unique reflections	58989	60461	53468
R_{work}/R_{free} (%)	17.7/21.1	19.3/22.7	19.7/23.1
No. non-H atoms			
Protein	2954	2951	2954
Ligand and ion	1	32	32
water	440	322	122
Average B factors (Å^2)			
Protein	29.20	31.78	36.74
Ligand and ion	18.21	90.88	99.04
water	37.25	37.03	36.58
R.m.s. deviations			
Bond lengths (Å)	0.011	0.007	0.008
Bond angles (°)	1.67	1.17	1.22

*Highest resolution shell (in Å) are shown in parentheses.

Supplementary Table 2. X-ray Data Collection and Structure Refinement Statistics

**Crystal**	apo hcGAS WT	hcGAS WT+cGAMP	hcGAS WT+pppGpG
PDB code	8SHZ	8SI0	8SJ8
**Data collection**			
Beamline	17-ID-1 (AMX)	17-ID-2 (FMX)	17-ID-1 (AMX)
Wavelength (Å)	0.9201	0.9793	0.9793
Space group	P 21 2 21	P 21 2 21	P 21 2 21
Cell constants			
a, b, c (Å)	47.90, 59.55, 124.82	47.93, 59.66, 125.33	47.77, 59.58, 124.75
α, β, γ (°)	90.00, 90.00, 90.00	90.00, 90.00, 90.00	90.00, 90.00, 90.00
Resolution (Å)	28.96 -2.40 (2.49-2.40) *	27.85-2.70 (2.79 -2.70) *	28.98-2.50 (2.59-2.50) *
R_{merge}	0.053	0.072	0.069
I/σ (I)	24.32 (10.10)	16.14 (7.36)	18.97 (8.77)
Completeness (%)	99.50 (94.8)	99.70 (96.9)	98.80 (95.90)
Redundancy	5.8 (5.1)	4.6 (4.1)	5.8 (5.1)
**Refinement**			
No. unique reflections	14467	10385	12635
R_{work}/R_{free} (%)	21.3/25.8	22.8/27.7	21.5/26.9
No. non-H atoms			
Protein	2756	2787	2685
Ligand and ion	1	46	56
water	11	2	7
Average B factors (Å^2)			
Protein	60.87	57.65	59.83
Ligand and ion	27.19	76.59	109.66
water	31.70	30.00	30.00
R.m.s. deviations			
Bond lengths (Å)	0.009	0.010	0.010
Bond angles (°)	1.23	1.32	1.33

*Highest resolution shell (in Å) are shown in parentheses.

Supplementary Table 3. X-ray Data Collection and Structure Refinement Statistics

**Crystal**	cGAS QN:dsDNA +ATP	cGAS WT:dsDNA +ATP/Mn	cGAS QN:dsDNA +GTP
PDB code	7UUX	8SKT	7UYQ
**Data collection**			
Beamline	17-ID-2	17-ID-2	17-ID-2
Wavelength (Å)	0.9793	1.8914	0.9793
Space group	P 21 21 21	P 21 21 21	P 21 21 21
Cell constants			
a, b, c (Å)	77.99 98.19 143.18	78.03 98.39 142.59	77.75, 99.58, 142.00
α, β, γ (°)	90.00, 90.00, 90.00	90.00, 90.00, 90.00	90.00, 90.00, 90.00
Resolution (Å)	29.53-2.26 (2.34-2.26) *	29.58-2.69 (2.78-2.69) *	28.91-2.57 (2.66-2.57) *
R_{merge}	0.067	0.109	0.091
I/σ (I)	16.11 (2.22)	12.63 (2.51)	13.71 (2.06)
Completeness (%)	99.72 (98.60)	99.55 (97.01)	99.36 (95.00)
Redundancy	6.8 (5.9)	6.6 (6.5)	6.7 (6.6)
**Refinement**			
No. unique reflections	52115	31210	35639
R_{work}/R_{free} (%)	20.90/24.00	19.87/24.68	20.13/23.16
No. non-H atoms			
Protein	7239	7204	7151
Ligand and ion	90	68	132
water	92	25	47
Average B factors (Å^2)			
Protein	63.62	60.02	61.27
Ligand and ion	40.10	44.00	58.69
water	47.47	44.13	42.66
R.m.s. deviations			
Bond lengths (Å)	0.011	0.009	0.009
Bond angles (°)	1.51	1.07	1.53

*Highest resolution shell (in Å) are shown in parentheses.

Supplementary Table 4. X-ray Data Collection and Structure Refinement Statistics

**Crystal**	cGAS QN:dsDNA +ATP/GTP	cGAS QN:dsDNA +ATP/ITP	cGAS QN:dsDNA +GTP/ITP
PDB code	7UXW	8G1J	8G10
**Data collection**			
Beamline	17-ID-2	17-ID-1	17-ID-1
Wavelength (Å)	0.9793	0.9201	0.9201
Space group	P 21 21 21	P 21 21 21	P 21 21 21
Cell constants			
a, b, c (Å)	78.93, 98.66, 142.25	78.09, 98.48, 142.10	78.01, 98.66, 142.28
α, β, γ (°)	90.00, 94.59, 90.00	90.00, 90.00, 90.00	90.00, 90.00, 90.00
Resolution (Å)	28.99-2.57 (2.66-2.57) *	29.60-2.29 (2.38-2.29) *	29.64-2.47 (2.56-2.47) *
R_{merge}	0.097	0.065	0.082
I/σ (I)	13.54 (2.08)	17.58 (2.28)	15.61 (2.22)
Completeness (%)	99.50 (96.30)	99.78 (98.91)	99.54 (96.92)
Redundancy	6.7 (6.6)	6.8 (6.8)	6.8 (6.7)
**Refinement**			
No. unique reflections	35971	49507	40107
R_{work}/R_{free} (%)	20.19/24.60	20.07/23.33	19.49/22.83
No. non-H atoms			
Protein	7176	7229	7230
Ligand and ion	130	128	130
water	39	104	87
Average B factors (Å^2)			
Protein	65.15	61.91	59.54
Ligand and ion	61.88	56.24	58.80
water	46.82	51.19	47.63
R.m.s. deviations			
Bond lengths (Å)	0.020	0.009	0.009
Bond angles (°)	1.65	1.26	1.32

*Highest resolution shell (in Å) are shown in parentheses.

Supplementary Table 5. X-ray Data Collection and Structure Refinement Statistics

**Crystal**	cGAS QN:dsDNA +pppGpA/Mn	cGAS WT:dsDNA +pppGpG/Mg	cGAS WT:dsDNA +pppGpG/Mn
PDB code	7V0W	7UYZ	7UZR
**Data collection**			
Beamline	17-ID-1	17-ID-1	17-ID-2
Wavelength (Å)	1.8785	0.9199	0.9793
Space group	P 21 21 21	P 21 21 21	P 21 21 21
Cell constants			
a, b, c (Å)	78.48, 99.47, 142.09	78.34, 98.52, 142.69	78.53, 99.00, 143.09
α, β, γ (°)	90.00, 90.00, 90.00	90.00, 90.00, 90.00	90.00, 90.00, 90.00
Resolution (Å)	28.91-2.66 (2.75-2.66) *	29.83-2.49 (2.58-2.49) *	28.99-2.69 (2.792-2.69) *
R_{merge}	0.096	0.144	0.118
I/σ (I)	12.04 (1.76)	10.04 (2.16)	12.17 (1.68)
Completeness (%)	99.43 (97.15)	99.27 (94.98)	98.90 (92.60)
Redundancy	6.5 (6.3)	6.9 (6.7)	6.6 (6.3)
**Refinement**			
No. unique reflections	32652	39182	31146
R_{work}/R_{free} (%)	20.53/24.15	21.78/24.77	19.39/24.23
No. non-H atoms			
Protein	7132	7170	7206
Ligand and ion	112	116	116
water	24	70	21
Average B factors ($Å^2$)			
Protein	75.59	56.45	66.86
Ligand and ion	65.18	55.81	67.96
water	53.00	37.88	41.10
R.m.s. deviations			
Bond lengths (Å)	0.010	0.009	0.011
Bond angles (°)	1.44	1.43	1.53

*Highest resolution shell (in Å) are shown in parentheses.

Supplementary Table 6. X-ray Data Collection and Structure Refinement Statistics

Crystal	cGAS WT:dsDNA +pppGpI	cGAS WT:dsDNA +pppIpA	cGAS:dsDNA +2d'ATP
PDB code	8EAE	8G23	8SJ0
**Data collection**			
Beamline	17-ID-1	17-ID-2	17-ID-1
Wavelength (Å)	0.9201	0.9793	0.9202
Space group	P 21 21 21	P 21 21 21	P 21 21 21
Cell constants			
a, b, c (Å)	77.43, 99.32, 141.72	77.72, 98.89, 142.14	77.50, 98.30, 143.21
α, β, γ (°)	90.00, 90.00, 90.00	90.00, 90.00, 90.00	90.00, 90.00, 90.00
Resolution (Å)	28.84-2.56 (2.65-2.57) *	29.68-2.71 (2.80-2.71) *	29.80-2.55 (2.65-2.55) *
R_{merge}	0.090	0.099	0.088
I/σ (I)	12.78 (2.10)	11.24 (1.75)	14.24 (2.05)
Completeness (%)	99.20 (94.20)	97.02 (91.37)	99.40 (96.10)
Redundancy	5.6 (5.5)	5.4 (5.4)	6.7 (6.3)
**Refinement**			
No. unique reflections	35822	30365	36058
R_{work}/R_{free} (%)	20.20/23.35	19.05/24.89	19.4/24.0
No. non-H atoms			
Protein	7292	7167	7293
Ligand and ion	112	88	86
water	59	59	34
Average B factors (Å^2)			
Protein	60.08	61.48	66.65
Ligand and ion	73.70	66.52	63.76
water	42.05	55.47	51.48
R.m.s. deviations			
Bond lengths (Å)	0.009	0.070	0.010
Bond angles (°)	1.38	1.65	1.30

*Highest resolution shell (in Å) are shown in parentheses.

Supplementary Table 7. X-ray Data Collection and Structure Refinement Statistics

Crystal	cGAS:dsDNA +3‘-dATP	cGAS:dsDNA +ATP/2'-dGTP	cGAS:dsDNA +ATP.10 mM Mg
PDB code	8SJ1	8SJ2	8GIM
**Data collection**			
Beamline	17-ID-1	17-ID-1	17-ID-2
Wavelength (Å)	0.9202	0.9201	1.8914
Space group	P 21 21 21	P 21 21 21	P 21 21 21
Cell constants			
a, b, c (Å)	77.18, 97.58, 142.28	78.63, 98.85, 142.78	77.90, 98.00, 142.47
α, β, γ (°)	90.00, 90.00, 90.00	90.00, 90.00, 90.00	90.0, 90.0, 90.0
Resolution (Å)	28.74-2.81 (2.91-2.81)*	29.72-2.23 (2.31-2.23)*	29.69-2.63 (2.73-2.63)*
R_{merge}	0.144	0.082	0.08
I/σ(I)	9.14 (2.17)	12.70 (2.50)	11.71 (2.61)
Completeness (%)	99.6 (97.4)	99.30 (96.60)	99.12 (94.42)
Redundancy	6.7 (6.0)	6.7 (6.0)	5.1 (4.8)
**Refinement**			
No. unique reflections	27032	54457	32701
R_{work}/R_{free} (%)	18.9/23.9	21.0/24.5	19.0/22.9
No. non-H atoms			
Protein	7294	7241	7294
Ligand and ion	64	128	68
water	16	115	22
Average B factors (Å^2)			
Protein	54.93	62.04	64.56
Ligand and ion	80.90	67.14	45.78
water	41.33	50.10	47.01
R.m.s. deviations			
Bond lengths (Å)	0.010	0.010	0.010
Bond angles (°)	1.33	1.21	1.29

*Highest resolution shell (in Å) are shown in parentheses.

Supplementary Table 8. X-ray Data Collection and Structure Refinement Statistics

Crystal	cGAS:dsDNA + ATP.10 mM Mg		
	15 µM Mn	40 µM Mn	100 µM Mn
PDB code	8GIN	8GIP	8GIO
**Data collection**			
Beamline	17-ID-2	17-ID-2	17-ID-2
Wavelength (Å)	1.8914	1.8914	1.8914
Space group	P 21 21 21	P 21 21 21	P 21 21 21
Cell constants			
a, b, c (Å)	77.39, 98.71, 142.26	77.93, 98.40, 142.47	77.59, 98.62, 141.93
α, β, γ (°)	90.0, 90.0, 90.0	90.0, 90.0, 90.0	90.0, 90.0, 90.0
Resolution (Å)	29.78-2.75 (2.85-2.75) *	29.79-2.70 (2.80-2.70) *	28.80-2.67 (2.76-2.67) *
R_{merge}	0.11	0.10	0.10
I/σ (I)	11.24 (2.93)	10.19 (2.57)	11.52 (2.95)
Completeness (%)	99.00 (97.60)	98.27 (95.80)	99.27 (94.29)
Redundancy	6.5 (6.1)	4.7 (4.3)	5.7 (5.2)
**Refinement**			
No. unique reflections	28657	30241	31527
R_{work}/R_{free} (%)	18.1/22.5	18.6/24.2	18.3/24.1
No. non-H atoms			
Protein	7294	7294	7235
Ligand and ion	68	68	68
water	21	40	25
Average B factors (Å^2)			
Protein	65.71	58.70	62.35
Ligand and ion	48.47	42.48	49.49
water	47.39	43.16	47.15
R.m.s. deviations			
Bond lengths (Å)	0.010	0.010	0.010
Bond angles (°)	1.27	1.28	1.26

*Highest resolution shell (in Å) are shown in parentheses.

Supplementary Table 9. X-ray Data Collection and Structure Refinement Statistics

Crystal	cGAS:dsDNA + ATP.10 mM Mg		
	200 μM Mn	500 μM Mn	1000 μM Mn
PDB code	8GIR	8GIS	8GIT
**Data collection**			
Beamline	17-ID-2	17-ID-2	17-ID-2
Wavelength (Å)	1.8914	1.8914	1.8914
Space group	P 21 21 21	P 21 21 21	P 21 21 21
Cell constants			
a, b, c (Å)	77.56, 98.51, 142.01	78.06, 98.22, 142.47	77.60, 98.21, 142.53
α, β, γ (°)	90.0, 90.0, 90.0	90.0, 90.0, 90.0	90.0, 90.0, 90.0
Resolution (Å)	29.79-2.50 (2.59-2.50) *	29.54-2.46 (2.55-2.46) *	29.22-2.72 (2.82-2.72) *
R_{merge}	0.09	0.10	0.10
I/σ (I)	12.24 (2.56)	10.62 (2.67)	12.61 (2.73)
Completeness (%)	99.32 (94.66)	99.31 (94.79)	99.56 (97.62)
Redundancy	6.5 (6.0)	5.6 (5.3)	6.6 (5.9)
**Refinement**			
No. unique reflections	38173	40386	29931
R_{work}/R_{free} (%)	19.1/22.9	19.2/28.3	18.8/23.4
No. non-H atoms			
Protein	7294	7294	7294
Ligand and ion	68	68	68
water	83	35	35
Average B factors (Å^2)			
Protein	57.61	60.04	61.16
Ligand and ion	43.22	44.50	47.72
water	44.62	43.67	45.88
R.m.s. deviations			
Bond lengths (Å)	0.009	0.010	0.010
Bond angles (°)	1.25	1.26	1.28

*Highest resolution shell (in Å) are shown in parentheses.

Supplementary Table 10. Tables summarizing the catalytic activity (NTase rates; enz^{-1} min^{-1}) of cGAS (200 nM) with 200 nM 60-bp dsDNA, and 200 μM (total) NTPs. $n = 3$, ± S.D.

	Substrates	NTase rate
	ATP/GTP	4.750 ± 0.376
WT-mcGAS^{cat}	ATP/ATP	0.054 ± 0.011
	GTP/GTP	0.280 ± 0.030
QN- mcGAS^{cat}	ATP/GTP	0.047 ±0.003
QN-hcGAS^{cat}	ATP/GTP	0.035 ± 0.030

	Substrates	NTase rate
	ATP/GTP	4.750 ± 0.376
	2'-dATP/GTP	0.693 ± 0.147
WT-mcGAS^{cat}	3'-dATP/GTP	0.656 ± 0.095
	ATP/2'-dGTP	0.104 ± 0.009

	Substrates	NTase rate
	ATP/GTP	4.750 ± 0.376
	ATP/ATP	0.054 ± 0.011
	GTP/GTP	0.280 ± 0.030
WT-mcGAS^{cat}	ATP/ITP	1.818 ± 0.126
	ITP/ITP	0.590 ± 0.066
	ATP/PuTP	0.134 ± 0.013
	PuTP/GTP	1.533 ± 0.226

	Substrates	NTase rate
	ATP/GTP	4.750 ± 0.376
WT-mcGAS^{cat}	ATP/ATP	0.054 ± 0.011
	GTP/GTP	0.280 ± 0.030
	ATP/GTP	0.799 ± 0.166
H467A-mcGAS^{cat}	ATP/ATP	0.043 ± 0.026
	GTP/GTP	0.453 ± 0.076

	Substrates	NTase rate
WT-mcGAS^{cat}	ATP/GTP	4.750 ± 0.376
	ATP/ATP	0.054 ± 0.011
	GTP/GTP	0.280 ± 0.030
R364A-mcGAS^{cat}	ATP/GTP	0.143 ± 0.012
	ATP/ATP	0.188 ± 0.042
	GTP/GTP	0.027 ± 0.020

	Substrates	NTase rate
WT-mcGAS^{FL}	ATP/GTP	8.679 ± 0.926
WT-mcGAS^{cat}	ATP/GTP	4.750 ± 0.376
WT-hcGAS^{FL}	ATP/GTP	0.498 ± 0.071
WT-hcGAS^{cat}	ATP/GTP	0.551 ± 0.088

	Substrates	NTase rate
WT-mcGAS^{cat}	ATP/GTP	4.750 ± 0.376
WT-hcGAS^{cat}	ATP/GTP	0.551 ± 0.088
T321I-hcGAS^{cat}	ATP/GTP	1.841 ± 0.125
T321I/S434C-hcGAS^{cat}	ATP/GTP	3.593 ± 0.423
WT-mcGAS^{cat}	ATP/ATP	0.054 ± 0.011
WT-hcGAS^{cat}	ATP/ATP	0.151 ± 0.022
T321I-hcGAS^{cat}	ATP/ATP	0.031 ± 0.004
WT-mcGAS^{cat}	GTP/GTP	0.280 ± 0.030
WT-hcGAS^{cat}	GTP/GTP	0.078 ± 0.014
T321I-hcGAS^{cat}	GTP/GTP	0.254 ± 0.022

Supplementary Table 11. Summary of ITC results. n = 3 ± S.D.

Related to Figure 2B-D				
**Syringe**	**Cell**	**K_D (μM)**	**ΔH (kcal/mol)**	**ΔS (cal/mol/deg)**
ATP	QN-mcGAS^{cat}	43.06±5.32	-4.92±0.33	5.53±1.58
ATP	QN-mcGAS^{cat} + dsDNA	14.94±1.01	-14.66±0.50	-26.93±4.32
GTP	QN-mcGAS^{cat}	11.37±2.21	-3.41±0.21	11.3±0.90
GTP	QN-mcGAS^{cat} + dsDNA	11.23±1.54	-12.49±0.78	-19.04±6.36
ATP/GTP	QN-mcGAS^{cat}	16.65±2.37	-4.35±0.24	5.76±0.73
ATP/GTP	QN-mcGAS^{cat} + dsDNA	5.15±0.96	-15.32±0.93	-27.1±2.00
ATP	QN-mcGAS^{cat} + GTP	N.B.	N.B.	N.B.
ATP	QN-mcGAS^{cat} + GTP + dsDNA	11.28±1.75	-3.69±0.26	10.31±1.99
GTP	QN-mcGAS^{cat} + ATP	40.04±6.05	-1.36±0.25	21.80±2.00
GTP	QN-mcGAS^{cat} + ATP + dsDNA	5.42±1.63	-2.44±0.20	16.3±1.2
cGAMP	QN-mcGAS^{cat}	16.37±3.49	-3.06±0.90	11.90±0.90
cGAMP	QN-mcGAS^{cat} + dsDNA	12.04±2.38	-4.95±1.57	8.97±0.52
ATP	K382E/QN-mcGAS^{cat}	40.74±9.04	-2.91±0.32	10.43±1.77
ATP	K382E/QN-mcGAS^{cat} + dsDNA	40.92±6.50	-3.22±0.45	8.29±2.31
GTP	K382E/QN-mcGAS^{cat}	12.59±2.42	-2.14±0.24	15.93±1.61
GTP	K382E/QN-mcGAS^{cat} + dsDNA	19.05±2.91	-2.19±0.14	14.4±0.2
ATP	K382E/QN-mcGAS^{cat} + dsDNA + GTP	N.B.	N.B.	N.B.
GTP	K382E/QN-mcGAS^{cat} + dsDNA + ATP	N.B.	N.B.	N.B.
**Related to Figure 3B**				
**Syringe**	**Cell**	**K_D (μM)**	**ΔH (kcal/mol)**	**ΔS (cal/mol/deg)**
2'-dATP	QN-mcGAS^{cat}	44.58±5.62	-8.84±0.71	-11.32±0.30
2'-dATP	QN-mcGAS^{cat} + dsDNA	33.64±3.15	-12.73±0.63	-17.21±4.91
3'-dATP	QN-mcGAS^{cat}	44.93±2.94	-9.67±0.33	-12.55±1.15
3'-dATP	QN-mcGAS^{cat} + dsDNA	41.68±3.76	-12.07±0.58	-16.95±0.85
GTP	QN-mcGAS^{cat} + 2'-dATP	118.17±20.40	-5.75±0.59	0.72±0.17
GTP	QN-mcGAS^{cat} + 3'-dATP	52.96±7.91	-8.01±0.76	-7.23±2.77
**Related to Figure 4D**				
**Syringe**	**Cell**	**K_D (μM)**	**ΔH (kcal/mol)**	**ΔS (cal/mol/deg)**
2'-dGTP	QN-mcGAS^{cat}	10.42±2.01	-3.05±0.17	12.6±2.3
2'-dGTP	QN-mcGAS^{cat} + dsDNA	7.68±2.07	-7.38±0.37	8.93±2.17
2'-dGTP	QN-mcGAS^{cat} + ATP	67.67±13.94	-2.37±0.31	11.22±2.97
2'-dGTP	QN-mcGAS^{cat} + ATP + dsDNA	63.62±7.26	-3.98±0.25	11.9±1.20
